# Anti-adhesive Properties of Epoxy-Treated Xenopericardium Modified with Polyvinyl Alcohol: *in vitro* Study of Leukocyte Adhesion in the Pulsatile Flow Model

**DOI:** 10.17691/stm2024.16.2.04

**Published:** 2024-04-27

**Authors:** E.A. Ovcharenko, T.V. Glushkova, D.K. Shishkova, M.A. Rezvova, E.A. Velikanova, K.Yu. Klyshnikov, T.N. Akentyeva, A.E. Kostyunin

**Affiliations:** PhD, Head of the Laboratory of New Biomaterials; Research Institute for Complex Issues of Cardiovascular Diseases, 6 Academician L.S. Barbarash Blvd, Kemerovo, 650002, Russia; PhD, Senior Researcher, Laboratory of New Biomaterials; Research Institute for Complex Issues of Cardiovascular Diseases, 6 Academician L.S. Barbarash Blvd, Kemerovo, 650002, Russia; PhD, Researcher, Laboratory of Molecular, Translational and Digital Medicine; Research Institute for Complex Issues of Cardiovascular Diseases, 6 Academician L.S. Barbarash Blvd, Kemerovo, 650002, Russia; Junior Researcher, Laboratory of New Biomaterials; Research Institute for Complex Issues of Cardiovascular Diseases, 6 Academician L.S. Barbarash Blvd, Kemerovo, 650002, Russia; PhD, Researcher, Laboratory of Cell Technologies; Research Institute for Complex Issues of Cardiovascular Diseases, 6 Academician L.S. Barbarash Blvd, Kemerovo, 650002, Russia; PhD, Researcher, Laboratory of New Biomaterials; Research Institute for Complex Issues of Cardiovascular Diseases, 6 Academician L.S. Barbarash Blvd, Kemerovo, 650002, Russia; Junior Researcher, Laboratory of New Biomaterials; Research Institute for Complex Issues of Cardiovascular Diseases, 6 Academician L.S. Barbarash Blvd, Kemerovo, 650002, Russia; PhD, Researcher, Laboratory of New Biomaterials; Research Institute for Complex Issues of Cardiovascular Diseases, 6 Academician L.S. Barbarash Blvd, Kemerovo, 650002, Russia

**Keywords:** bioprosthetic heart valves, polyvinyl alcohol, immune rejection, leukocytes, adhesion

## Abstract

**The aim of the study** is to assess protective capabilities of the polymer coating made of polyvinyl alcohol to prevent leukocyte adhesion to epoxy-treated bovine pericardium, which is used in production of bioprosthetic heart valves.

**Materials and Methods:**

Fragments of unmodified (control) and modified with polyvinyl alcohol epoxy-treated bovine pericardium were incubated in the dedicated chambers connected to a pulsatile flow system (Ibidi GmbH, Germany). During 48 h incubation was conducted in whole donor plasma containing 3**·**10^6^ of mononuclear fraction cells. To simulate plasma flow, the shear stress on the inflow and outflow sides of of bioprosthetic heart valve in the aortic position was set to 50 and 20 dynes/cm^2^, respectively. After the experiment was completed, the surface of the studied samples was subjected to scanning electron microscopy and immunofluorescence using antibodies to the pan-leukocyte marker CD45.

**Results:**

Adhesion of leukocytes (CD45^+^) was seen for both the serous (outflow side) and fibrous (inflow side) surfaces of the control epoxy-treated bovine pericardium, whereas both surfaces of the material modified with polyvinyl alcohol were clear of immune cells. Scanning electron microscopy confirmed the adhesion of leukocytes to intact biological tissue: the cells on the surface of the control xenopericardium were of an irregular shape and formed numerous pseudopodia.

**Conclusion:**

The suggested modification of epoxy-treated bovine pericardium with polyvinyl alcohol prevents the adhesion of immune cells to the implant surface and can potentially protect bioprosthetic heart valves from immune rejection.

## Introduction

In recent decades, bioprosthetic heart valves (BHVs) made from chemically stabilized animal tissues (porcine aortic valves, bovine or porcine pericardium) have been increasingly used to replace incompetent heart valves [1]. BHVs are characterized by low thrombogenicity and do not require lifelong administration of anticoagulants by their recipients [2]. At the same time, due to development of structural valve degeneration (SVD) the durability of BHVs is limited to an average of 15 years [3]. The specified disadvantage limits the possibilities of using BHVs in patients under 65 years, whose life expectancy exceeds the average durability of implants [4].

One of the main causes of SVD is the cell-mediated inflammatory [5]. Immune cells (neutrophils, macrophages and giant multinucleated cells) invade the BHV leaflets, which results in accumulation of the matrix-destructive (matrix metalloproteinases and cathepsins) and calcifying (osteopontin, osteocalcin and bone sialoprotein) factors produced by these cells [6–9]. Interaction of the mentioned substances with the BHV tissue leads to fragmentation and mineralization of its collagen basis. Despite the improved approaches to biomaterial processing aimed at reducing its immunogenicity, the problem of immune rejection of BHVs has not been solved yet [5].

The most promising current approach to eliminate the negative influence of the recipient factors (including immune cells) on the BHVs tissue is the tissue treatment with biocompatible polymers [10, 11]. Such polymers are to form hydrogel in the interfibrillar space of the biomaterial, as well as to form a protective film on its surface, preventing imbibition of substances dissolved in the blood and invasion of immune cells.

Previously, the authors developed an original modification of epoxy-treated bovine pericardium with polyvinyl alcohol, which significantly increased the biomaterial resistance to calcification without deterioration of its mechanical and hemocompatible properties [11]. This modification provides for impregnation of biological tissue with liquid polyvinyl alcohol and further polymerization of the tissue by cryostructuring. The procedure results in production of a composite material being a collagen fiber matrix filled with hydrogel [11]. In this study, the authors assess the possibility of using this approach to protect biomaterials from immune cell adhesion. For this purpose, the researchers developed an *in vitro* model that allows creating a laminar flow of plasma containing living cells on the surface of the sample.

**The aim of the study.** In this study, the authors set two goals. The main goal was to evaluate the protective properties of the polyvinyl alcohol coating used to prevent the immune cell adhesion to epoxy-treated bovine pericardium involved in production of BHVs. The supplement goal was to test an original *in vitro* model that simulates the whole blood flow washing the BHV leaflets in aortic position.

## Materials and Methods

### Studied biomaterial

As the material for the study the authors used fragments of KemPeriplas-Neo (KPi7080M; NeoCor, Russia) bovine pericardial patch with a thickness of 0.6–0.7 mm, modified with a 12% aqueous solution of polyvinyl alcohol in accordance with the original technique developed by the authors [11]. The control group consisted of unchanged bovine pericardial patch.

### Simulation of the plasma flow washing the bioprosthetic heart valve leaflets

**Figure 1.**
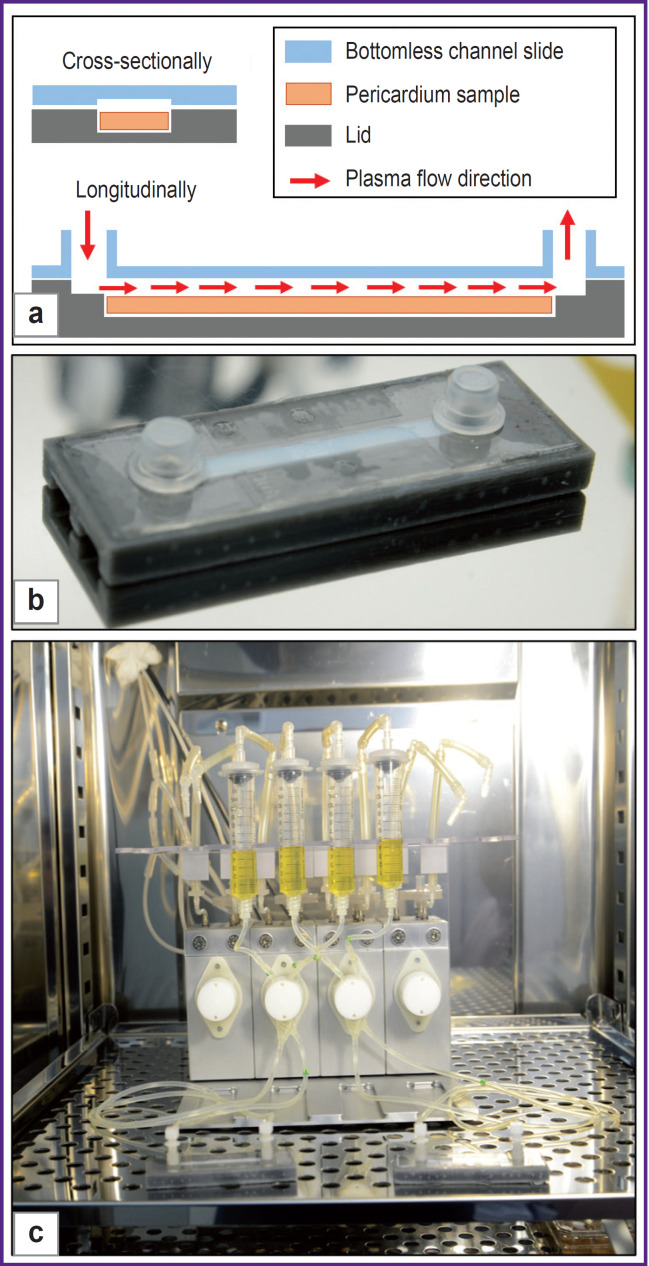
Design of chamber creating the laminar plasma flow on the sample surface: (a) chamber layout; (b) chamber appearance; (c) chambers connected to the pulsatile flow system

To study the adhesive properties of epoxy-treated bovine pericardium modified with polyvinyl alcohol, the authors developed a special chamber (Figure 1) that allows to create a laminar flow of plasma containing living cells (leukocytes) on the surface of the sample. For this purpose, the authors applied bottomless channel slides (80168; Ibidi GmbH, Germany), the bottom self-adhesive surface of which was attached with sterile customized polylactide lids (002863; REC, Russia). These lids were made layer-by-layer using the Ender-3 S1 PRO 3D printer (Creality, China). Biomaterial samples were placed in a groove at the bottom of the lid, and the chamber was connected to the Ibidi Pump System Quad pulsatile flow system (Ibidi GmbH, Germany) using the appropriate Perfusion Set Yellow/Green connectors (10964; Ibidi GmbH, Germany). One should note that the equipment in this study (including the pulsatile flow system and the bottomless channel slides) is typically used to simulate *in vivo* conditions for culturing cells in flow (for example, endothelial cells of blood and lymphatic vessels) [12, 13]. Due to the channel slide lids made by the authors, the system was adjusted to the study objectives.

To simulate the flow on the inflow side of the BHV in the aortic position, the shear stress was set to 50 dynes/ cm^2^, whereas on the outflow side — 20 dynes/cm^2^ [14, 15]. 15 ml of donor plasma was used for each module of the flow system. Simultaneously, the researchers connected two chambers with a modified and unchanged xenopericardium, located on the flow of the fibrous (simulation of the BHV inflow side) or serous (simulation of the BHV outflow side) surface. The said construction was placed in a carbon-dioxide incubator at 37°C. The study samples were exposed for 48 h. All experiments were conducted using the blood and leukocytes of one conditionally healthy volunteer.

Before starting the work with the pulsatile flow system, the researchers prepared 60 ml of plasma obtained by centrifuging whole blood for 10 min at 2000 g. This plasma was stored at –80°C until used.

Leukocytes were isolated from the blood by sedimentation in the Ficoll gradient with a density of 1.077 g/cm^3^ (P053; PanEco, Russia). For this purpose, whole blood was diluted in the 1:1 ratio with Dulbecco’s phosphate-buffered saline solution void of calcium and magnesium (1.2.4.7; BioloT, Russia). The diluted blood was layered onto the Ficoll solution having 1.5 ml of blood per 1 ml of solution and centrifuged for 30 min at 400 g and room temperature. Then the mononuclear fraction of blood cells that formed a whitish interphase at the interface between the Ficoll solution and plasma, was transferred to a clean vial and washed twice with Dulbecco’s buffer solution for 10 min at 350 g, and then for another 10 min at 300 g and room temperature. Finally, the leukocyte sediment was dissolved in the prepared plasma with 3**·**10^6^ cells per one module of the flow system.

### Immunofluorescent analysis

Cells adhering to the studied xenopericardium samples were detected using immunofluorescent analysis. For this purpose, biomaterial fragments were fixed for 10 min in 4% paraformaldehyde at room temperature and twice washed (10 min each wash) in phosphate-buffered saline (pH 7.4) on the Polymax 1040 shaker (Heidolph, Germany) at 50 rpm. Blocking of nonspecific antibody binding was performed by incubation of samples in 1% bovine serum albumin for 1 h. Primary antibodies to the pan-leukocyte marker CD45 (ab10558; Abcam, UK) were diluted in 1% bovine serum albumin at a ratio of 1:1500. Then the test samples were placed into the solution, where they were incubated for 20 h at 4ºC. After incubation of the samples in the solution with primary antibodies, they were twice washed in the phosphatebuffered saline (10 min each wash) and incubated in the 1% solution of bovine serum albumin added with secondary anti-rabbit antibodies (ab150074; Abcam, UK; dilution 1:500), conjugated with Alexa Fluor 555 fluorescent labels (Thermo Fisher Scientific, USA). After being twice washed in the phosphate-buffered saline, the samples were treated with 70% ethyl alcohol for 5 min and with autofluorescence elimination reagent (2160; Merck, Germany) for another 5 min. After being washed three times in 70% ethanol (1 min each wash, on a shaker), tissue fragments were incubated in the solution of 4’,6-diamidino-2-phenylindole (0.1 μg/ml) for 30 min. Then, they were twice washed in the phosphate-buffered saline and once in the double-distilled water (10 min each wash); here, the samples were fixed on glass slides for further study using the LSM 700 laser scanning confocal microscope (Carl Zeiss, Germany).

Image processing was conducted using the Zen software (Carl Zeiss, Germany). To semi-quantitatively estimate adherent leukocytes, cell counts were performed in 15 randomly selected fields of view at 200× magnification.

### Scanning electron microscopy

To confirm the leukocyte adhesion to the studied biomaterial, scanning electron microscopy was used. For this purpose, the samples were first washed from unadherent cells in the phosphate-buffered saline solution (3 times for 5 min), then fixed in 2% glutaraldehyde overnight. Next, the samples were washed from glutaraldehyde in the phosphate-buffered saline (3 times for 15 min) and once in the double-distilled water (5 min). Further, the samples were frozen at –40°C and lyophilized using the FreeZone 2.5 Plus machine (Labconco Corporation, USA) during 24 h. Then the samples were fixed on special stages and the authors formed conductive coatings (Au/Pd) on their surface using the EM ACE200 system (Leica Microsystems, Germany). The samples surface structure was studied with the S-3400N scanning electron microscope (Hitachi, Japan) under high vacuum subject to accelerating voltage of 10 kV in secondary electron mode.

### Statistical analysis

Statistical data processing was conducted using the GraphPad Prism 8 software (GraphPad Software, USA). The data distribution type was determined using the Kolmogorov–Smirnov test. The distribution in the groups was not normal, thus the data are given as median and quartiles [Q1; Q3], as well as minimum and maximum values (min–max). Intergroup comparisons were made using the Kruskal–Wallis test adjusted for multiple comparisons (FDR). Intergroup differences were considered statistically significant with the maximum permissible probability of rejecting the correct null hypothesis p<0.05.

## Results and Discussion

**Figure 2.**
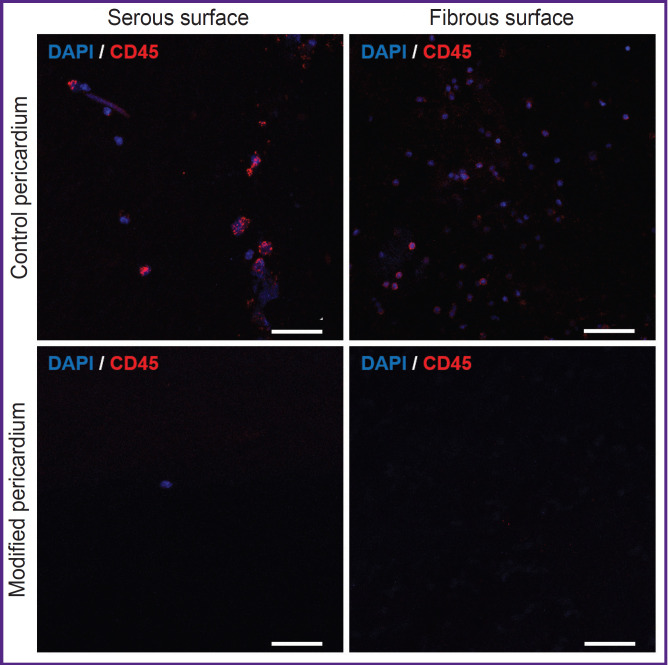
Visualization of leukocytes (CD45^+^) on the surface of the control and modified with polyvinyl alcohol xenopericardium. Bar — 50 μm

**Figure 3.**
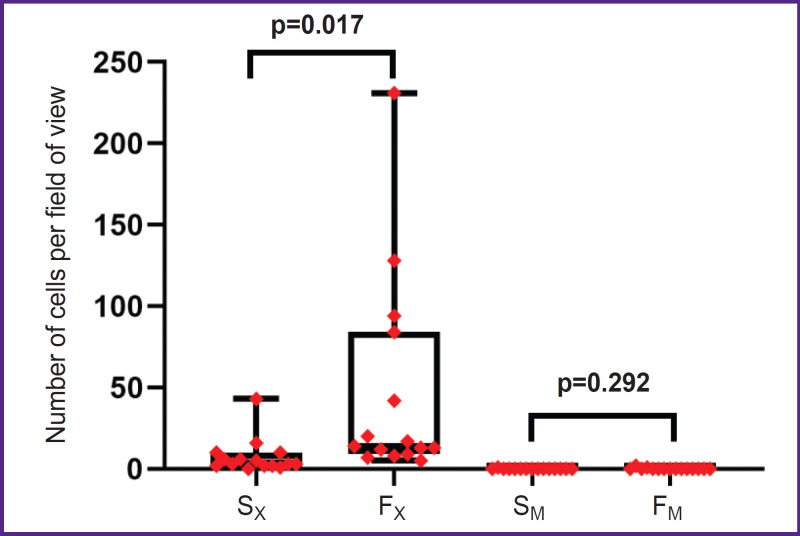
Semi-quantitative estimation of leukocytes adhering to the studied samples: S_X_ and F_X_ are the serous and fibrous surfaces of unmodified epoxy-treated xenopericardium, respectively (control); S_M_ and F_M_ are the serous and fibrous surfaces of the xenopericardium modified with polyvinyl alcohol, respectively

Analysis of the studied biomaterial samples by the immunofluorescence method demonstrated leukocyte accumulations (CD45^+^) on both surfaces of the control epoxy-treated bovine pericardium (Figure 2). Semi-quantitative estimation showed the statistically significant fact that more leukocytes sedimented on the hairy fibrous surface of the control xenopericardium than on the smooth serous surface (14 [9; 84], 5–231 vs. 3 [2; 10], 0–43, respectively, p=0.017). At that, there were practically no leukocytes on the surface of the samples treated with polyvinyl alcohol. Inventory of adherent cells did not reveal differences between the serous and fibrous surfaces of the modified pericardium (0 [0; 0], 0–1 and 0 [0; 0], 0–2, respectively, p=0.292), but the biomaterial samples in the experimental group differed sharply in this indicator from the control samples (p<0.0002; Figure 3).

**Figure 4.**
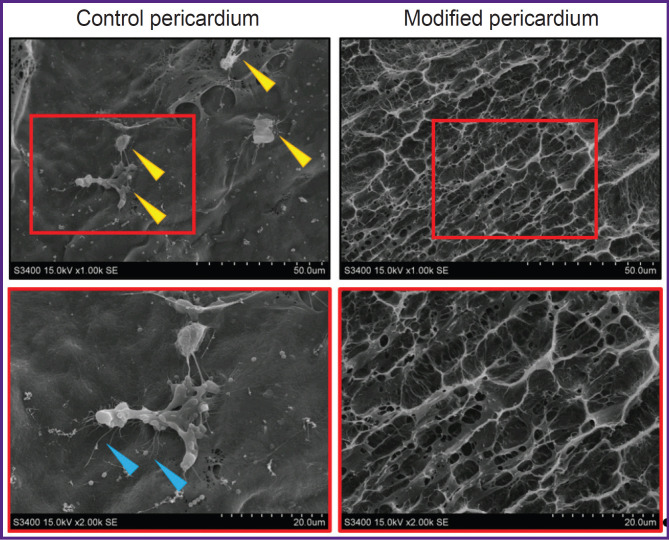
Electron microscopy of the surface of the control and modified with polyvinyl alcohol xenopericardium Numerous leukocyte pseudopodia show cell adhesion to the biomaterial. *Yellow arrows* — immune cells, *blue arrows* — leukocyte pseudopodia. The images in the lower row are enlarged images from the upper row

Data obtained by means of scanning electron microscopy confirmed the leukocytes adhesion to unmodified (control) bovine pericardium: the cells found on the surface of the biomaterial were of an irregular shape and formed numerous pseudopodia (Figure 4). Conversely, no immune cells were found on xenopericardial fragments treated with polyvinyl alcohol; the sample surface had a fine-mesh structure typical of a hydrogel [11].

In general, the results obtained indicate the excellent protective properties of the coating made of polymerized polyvinyl alcohol: as hydrogel has no cell adhesion sites, leukocytes cannot attach to the surface of the modified biomaterial and, thus, penetrate into it. Hence, the modification suggested by authors has the potential to solve the problem of vulnerability of current BHVs to infiltration of immune cells, which is crucial for increasing the durability of the implants. However, additional *in vivo* tests on large laboratory animals (sheep or pigs) are required to confirm these findings.

One should note that the available literature does not provide information about the suitability of the equipment used in this study for studying adhesion of leukocytes and other cells (for example, platelets, circulating stem cells) on various materials, possibly due to the lack of appropriate commercial chambers for test samples. The authors’ experience with customized lids shows the potential of this approach and significantly expands the scope of *in vitro* experimental modeling.

## Conclusion

The results of the studies demonstrated that modification of epoxy-treated bovine pericardium with polyvinyl alcohol allows to avoid adhesion of circulating immune cells to the BHV leaflets. The modified biomaterial is protected from leukocyte invasion and immune rejection. Potentially, the proposed modification may be useful for the development of the next-generation BHVs that are more resistant to SVD compared to the currently available models.

The authors developed lids, which were made of polylactide and were compatible with bottomless channel slides and the Ibidi Pump System Quad pulsatile flow system (Ibidi GmbH, Germany); these lids allow testing various materials for adhesion of living cells (leukocytes, platelets, stem cells) circulating in the blood. This is the first time this approach is described.
